# Investigation of prevalence of free Shiga toxin-producing *Escherichia coli* (STEC)-specific bacteriophages and its correlation with STEC bacterial hosts in a produce-growing area in Salinas, California

**DOI:** 10.1371/journal.pone.0190534

**Published:** 2018-01-04

**Authors:** Yen-Te Liao, Irwin A. Quintela, Kimberly Nguyen, Alexandra Salvador, Michael B. Cooley, Vivian C. H. Wu

**Affiliations:** 1 Produce Safety and Microbiology Research Unit, U.S. Department of Agriculture, Agricultural Research Service, Western Regional Research Center, Albany, California, United States of America; 2 School of Food and Agriculture, University of Maine, Orono, Maine, United States of America; USDA-ARS Salinity Laboratory, UNITED STATES

## Abstract

Shiga toxin-producing *E*. *coli* (STEC) causes approximately 265,000 illnesses and 3,600 hospitalizations annually and is highly associated with animal contamination due to the natural reservoir of ruminant gastrointestinal tracts. Free STEC-specific bacteriophages against STEC strains are also commonly isolated from fecal-contaminated environment. Previous studies have evaluated the correlation between the prevalence of STEC-specific bacteriophages and STEC strains to improve animal-associated environment. However, the similar information regarding free STEC-specific bacteriophages prevalence in produce growing area is lacking. Thus, the objectives of this research were to determine the prevalence of STEC-specific phages, analyze potential effects of environmental factors on the prevalence of the phages, and study correlations between STEC-specific bacteriophages and the bacterial hosts in pre-harvest produce environment. Surface water from 20 samples sites was subjected to free bacteriophage isolation using host strains of both generic *E*. *coli* and STEC (O157, six non-O157 and one O179 strains) cocktails, and isolation of O157 and non-O157 STEC strains by use of culture methods combined with PCR-based confirmation. The weather data were obtained from weather station website. Free O145- and O179-specific bacteriophages were the two most frequently isolated bacteriophages among all (O45, O145, O157 and O179) in this study. The results showed June and July had relatively high prevalence of overall STEC-specific bacteriophages with minimum isolation of STEC strains. In addition, the bacteriophages were likely isolated in the area—around or within city—with predominant human impact, whereas the STEC bacterial isolates were commonly found in agriculture impact environment. Furthermore, there was a trend that the sample sites with positive of free STEC bacteriophage did not have the specific STEC bacterial hosts. The findings of the study enable us to understand the ecology between free STEC-specific phages and STEC bacteria for further pre-harvest food safety management in produce environment.

## Introduction

Shiga toxin-producing *E*. *coli* (STEC) strains are foodborne pathogens that can cause serious human illness, such as hemolytic uremic syndrome and thrombotic thrombocytopenic purpura. High mortality is usually found in the population of the elders and children under 5 years of age. Because ruminants’ gastrointestinal tracts are the reservoir for the pathogens, the diseases primarily developed through consumption of undercooked and tenderized meat, particular beef products [1]. Nevertheless, during fall 2006, a foodborne outbreak associated with consumption of contaminated spinach occurred in several states. More than 200 victims were diagnosed with infection of *E*. *coli* O157:H7, and 3 people died [2]. In 2011, a catastrophic outbreak occurred in Germany associated with STEC contamination on fenugreek sprouts, and more than 3,000 cases of human infection were recorded with 850 patients developing HUS and 53 died [3]. Ever since the concerns regarding STEC infection has been raised on the produce safety. Produce contamination is a particularly serious situation since it is generally consumed raw.

Although produce can be easily contaminated at any point where pathogens are present in the production line, surface water, such as rivers or lakes, as a starting source of contamination in pre-harvest environment, is responsible for the spread of the various foodborne pathogens, including STEC, to produce through irrigation. Surface water also leads to infection of wildlife, which may lead to produce contamination due to deposition of feces in the field [4]. Wildlife, runoff from domestic animal facilities, and sewage are the major sources contributing to contamination of surface water [5]. In the meanwhile, bacteriophages (or phages) that are lytic against enteric bacteria, such as STEC, were also found to be key elements of intestinal microbiota of humans and other animals [6, 7]. Previous studies isolated free bacteriophages against *E*. *coli* (also known as coliphages) or against STEC strains (or STEC-specific phages) from the environments highly contaminated with feces of human or animal origins. For example, previous research focused on isolation of free STEC-specific phages, including serogroups of O26, O111, and O157, from cattle manure [8]. Their findings indicated that these phages were free of *stx*, *hlyA*, and *eaeA* genes and were good candidates as bio-control agents to prevent STEC contamination. The research conducted by Muniesa and Jofre with regard to prevalence of the bacteriophages against *E*. *coli* O157:H7 in sewage contaminated with fecal matters showed that the free phages infecting *E*. *coli* O157:H7 strains also harbored *stx2* genes and were commonly found in the sewage collected from different European countries [9]. Imamovic et al. evaluated different sources of water with regard to the prevalence of coliphages encoding *stx2* genes (or Stx2 phages) and found that the phages were positive in 70% and 90% of urban sewage and animal wastewater samples, respectively [10]. In addition, the authors indicated that these free Stx phages might pose a risk for the spread of *stx* genes among bacterial populations, and could result in emergence of new pathogens. These research findings indicate that there were various coliphages or STEC-specific phages in the animal-associated environment, and each type of phage might have different roles with regard to the bacterial hosts and the environment. However, similar information is lacking with regard to the produce production environment.

A study was conducted by U.S. Department of Agriculture research team to evaluate the prevalence of STEC strains and other foodborne pathogens and the persistence of pathogen subtypes in a produce central area [11]. The findings demonstrated that foodborne pathogens, including STEC were introduced into produce pre-harvest environment from wildlife and animal operations and were likely disseminated through surface water. Recently, the same research team also conducted another study using male-specific coliphages, one group of *E*. *coli* infecting phages, to track the source of fecal contamination from surface water in proximity to produce-intense regions [12]. Their findings could not correlate fecal pollution to the prevalence of male-specific phages, nor did it provide any information regarding whether or not the phages harbored *stx* genes, and its host range. In addition, the results showed that both non-O157 STEC and O157 STEC strains were isolated in the environment; however, the prevalence of free phages against O157 and non-O157 STEC was not determined [12]. So far, no published study aiming at the prevalence of free STEC-specific phages in produce-growing area is found. There is also a need to evaluate the potential environmental factors that may affect the prevalence free STEC-specific phages and to determine its association with STEC strains in pre-harvest produce area in order to improve safety of produce-growing area. Therefore, the objectives of this research were to 1) determine the prevalence of STEC-specific phages, to *2)* analyze potential effects of environmental factors on the prevalence of the phages, and to 3) study correlations between STEC-specific bacteriophages and the bacterial hosts in pre-harvest produce environment.

## Materials and methods

### Sampling area and sample collection

**Fig 1:**
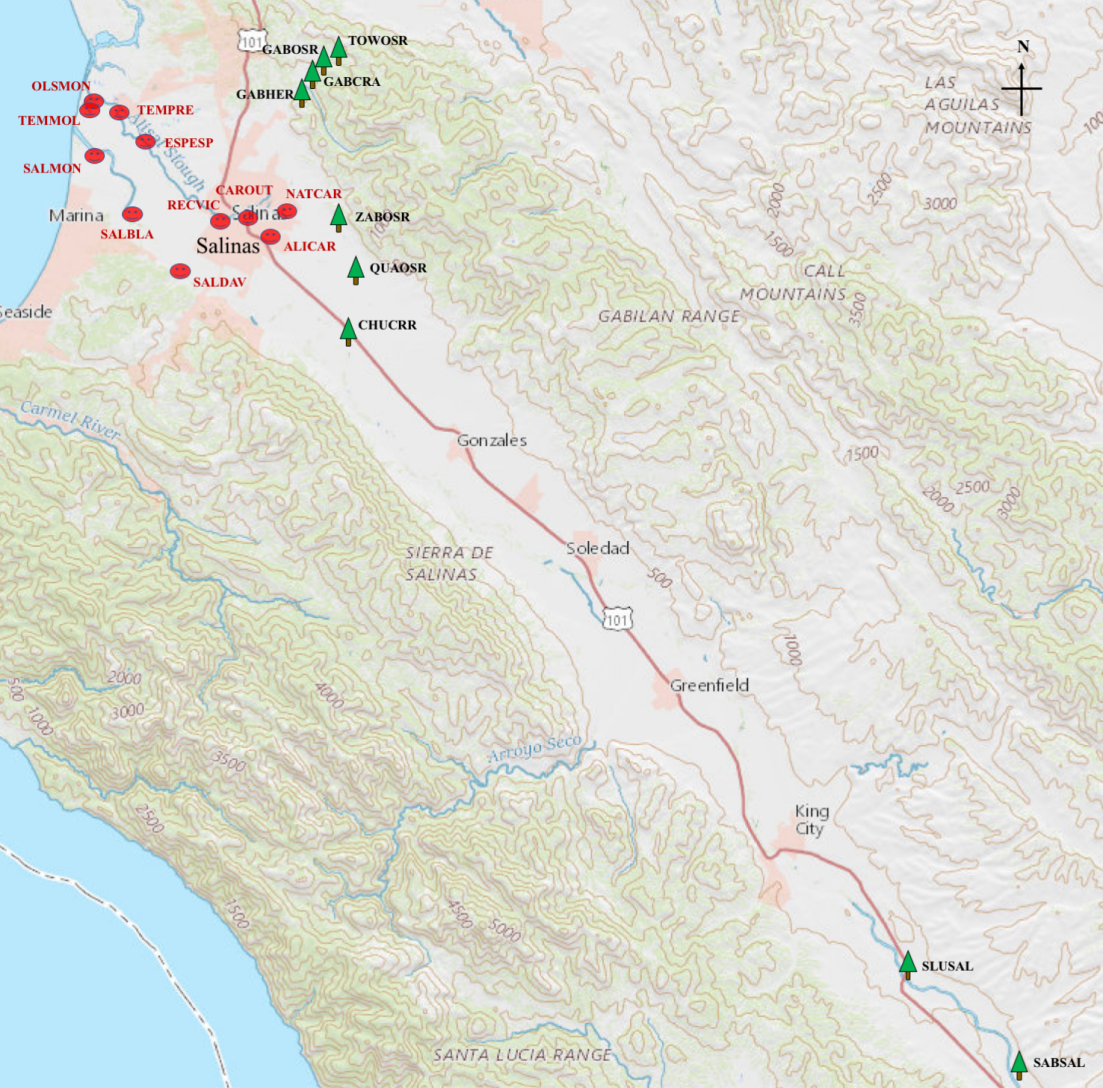
Geographical location of the watershed sites where the samples were collected in the area of Salinas Valley. Sample sites are labeled with a six-letter acronym in black type (locations with agriculture impact) or red type (locations with human impact). Insert is an expanded view of the area near the city of Salinas.

The sample sites within a heavy crop-growing area in Salinas Valley, CA were selected based on accessibility and previous experience. A total of 131 samples from 20 watershed sites were processed and categorized into several groups based on the potential for impact by human or agriculture activities. The sample sites labeled black acronym on the map indicated predominant agriculture impact and those with red acronym labeling indicated human impact in Fig 1. Since produce was grown throughout the Salinas valley, all 20 sample sites are impacted by agriculture, to a degree. However, sites SABSAL, SLUSAL and CHUCRR were located furthest up stream, in or near the Salinas River and sites QUAOSR, ZABOSR, TOWOSR, GABOSR, GABCRA, and GABHER were up stream, along the eastern side of the valley and in several tributaries feeding Carr Lake, within the city of Salinas. Importantly, cow calf operations were frequently observed on the hills above these sites. Thus, these sites were classified as agriculture-impacted sites. In contrast, the sites NATCAR, ALICAR, CAROUT, and RECVIC were located in Salinas City and were classified as human impact because of direct impact by human activities. Although the sites ESPESP, TEMPRE, TEMMOL, and OLSMON were along the Tembladero and Espinosa Sloughs near Castroville, where crop farms were located, the areas were also classified as human-impacted due to their location, downstream from Salinas and Castroville. Likewise, sites SALDAV, SALBLA, and SALMON, downstream on the Salinas River, were also classified as human impact due to runoff from several smaller townships along El Toro Creek and the Salinas River.

The samples collected from May to September 2016 were used for the isolation of both free STEC-specific phages and STEC strains. Water samples were collected using Moore swabs submerged in surface water for approximately 24h to collect sediment and microorganisms flowing through the swabs. The swab samples were placed in sterile sampling bags and stored on ice during transport to the ARS laboratory at Albany, CA. Due to lack of water, not every site was sampled during the sampling interval. Upon arrival, the swabs were added to 500 ml sterile water, followed by homogenization by hand for 20s. An aliquot of water (20mL) eluted from the swabs was stored at 4°C prior to further phage analysis. The majority of the sample (300mL), including the swab and debris, was used for STEC bacterial isolation. The remainder was used to isolate other pathogens in research not described here.

### STEC strain isolation

The STEC strains isolation protocol used in this study was described previously [13]. In brief, the swab including rinsate was enriched with TSB at 25°C for 2h and 42°C for 8h. For O157 STEC, the enrichment was subjected to immunomagnetic separation (IMS), followed by plating on selective media, Sorbital MacConkey (Difco Labs: Detroit, MI) with cefixine (0.05μg/mL; Introgen/Dynal) and tellurite (2.5μg/mL; Introgen/Dynal) (CT-SMAC) and Rainbow Agar O157 (Biolog, Hayward, CA) containing novobiocin (20μg/mL; Sigma-Aldrich) and tellurite (0.8μg/mL; Introgen/Dynal) (NT-RA), for isolation. Subsequently, the presumptive colonies were screened for the presence of *rfbE* gene by polymerase chain reaction (PCR) assay. As for non-O157 STEC, similar methods were used, including IMS, for isolation. A parallel isolation method included PCR screening for *stx* genes and was conducted immediately after sample enrichment. Subsequently, the *stx*-positive enrichments were plated on CHROMagar O157 (DRG International, Mountainside, New Jersey). The presumptive colonies were then confirmed for the presence of *stx* genes by PCR. The serotypes of the isolated STEC strains were determined by enzyme-linked immunosorbent assay (ELISA), and confirmed by *E*. *coli* Reference Center at Penn State University.

### Bacterial host strains for phage isolation

Four *E*. *coli* strains lacking *stx1* and *stx2* genes, including ATCC13706, ATCC43888, DH5, and MG1655, obtained from U.S. Department of Agriculture (USDA) Agricultural Research Service (ARS) Western Regional Research Center (WRRC), were used for isolating bacteriophage harboring *stx* genes. Four strains per serogroup of O26, O45, O103, O111, O121, O145 and O157, as well as one O179 strain previously isolated by USDA ARS WRRC, were also used for phage isolation, as well as host range test (Table 1). Some STEC strains previously isolated from water samples in Salinas Area were used to increase free phage isolation, and some isolated from different environmental samples, such as cattle feces and trough water, were also used as hosts to increase the variety of strains in this study. The overnight cultures of the selected host strains—non-pathogenic *E*. *coli* and STEC—were prepared by inoculating each sterile tube containing 5ml of tryptic soy broth (TSB, Difco, Becton Dickinson, Sparks, MD) with a 1μl-loop of the individual strain culture and incubating overnight at 37°C. The strain cocktail for either non-pathogenic or STEC strains was prepared by mixing 0.1ml of each overnight culture immediately prior to use. Both cocktails of non-pathogenic *E*. *coli* and STEC were used separately to inoculate the water samples for the isolation of free STEC-specific phages.

**Table 1:** Shiga toxin-producing *E*. *coli* (STEC) strains isolated from different sources by U.S. Department of Agriculture ARS used for free STEC-specific phage isolation.

ID# (RM)	Source	O type	H type	*eae*	*stx1*	*stx2*
17857	water	26	18	-	+	-
18118	water	26	-	+	+	-
18132	water	26	-	+	+	-
17133	water	26	-	-	+	-
12551	water	103	2	+	+	-
13322	cattle feces	103	2	+	+	-
8356	water	103	-	-	-	+
10744	cattle feces	103	-	+	-	+
10046	cattle feces	121	19	+	-	+
10068	trough water	121	19	+	-	+
8082	cattle feces	121	-	-	+	+
9982	water	121	-	+	-	+
13483	cattle feces	111	2	+	+	-
13789	water	111	-	+	+	-
11765	water	111	-	+	+	-
14488	water	111	-	+	-	+
8732	water	145	+	+	+	-
11691	water	145	+	+	+	-
12367	water	145	+	+	-	+
10808	cattle feces	145	-	+	+	-
10729	cattle	45	-	-	+	-
13726	cattle	45	-	-	+	-
13745	cattle	45	-	-	+	-
13752	cattle	45	-	-	+	-
18959	water	157	7	+	-	+
18961	water	157	7	+	-	+
18972	water	157	7	+	+	+
18974	water	157	7	+	-	+
13543	trough water	179	-	-	-	+

### Phage isolation and purification

Water samples were centrifuged at 4,000xg for 15min to get rid of sediments. An aliquot of 10ml water sample was added into two sterile tubes with 30ml TSB supplemented with 5mM CaCl_2_, and subsequently inoculated with 2.8ml and 0.4ml of respective STEC and non-pathogenic *E*. *coli* cocktails, respectively. The sample-culture mixers were left at room temperature for 15min prior to incubation in a bench top incubator with 150rpm shaking (Sheldon Manufacturing, Inc., Cornelius, OR) at 37°C for 48h. After incubation, chloroform at 4% v/v concentration was added into each enrichment tube and homogenized by use of a multi-purpose tube rotator (Thermo Fisher Scientific, Waltham, MA) at room temperature for 30min to kill both bacterial hosts and background flora. The supernatants were obtained after centrifugation at 8,000xg for 10min.

For confirming the presence of free STEC-specific phages and its host specificity, spot assay was conducted with the supernatants obtained from the enrichments on the selected host strains (STEC and non-pathogenic *E*. *coli*). Briefly, an aliquot of 0.2ml bacterial overnight culture was mixed with 10ml molten tryptic soy agar (TSA, Difco, Becton Dickinson, Sparks, MD) amended with 5mM Calcium Chloride (CaCl_2_) to enhance phage adsorption onto bacterial surface and poured into a sterile Petri plate. Subsequently, ten microliters of each supernatant were spotted on the strain-inoculated TSA plate and incubated at 37°C for 24h. As a result, the positive samples showed a circular clearing zone, indicating the presence of phages lytic against the tested strain. The supernatants, which were used for spot assay and had positive results, were further enriched with each of the spot test-positive strains by adding 0.1ml of supernatant with 0.5ml of the overnight bacterial culture supplemented with CaCl_2_ at 5mM in 5ml of TSB and incubated at 37°C for 24h. After incubation, the propagated phage solutions (also known as lysates) were centrifuged at 8,000xg for 10min to get rid of bacterial debris. Subsequently, the phage lysates were subjected to single layer plaque assay for phage separation. Briefly, the phage lysate was serial diluted, followed by mixing 0.2ml of the diluted lysate and 0.3ml overnight bacterial culture in 10ml molten TSA supplemented with CaCl_2_ prior to pouring to a sterile Petri dish plate. After incubation at 37°C for 24h, three segregated plaques with different sizes were selected for further purification. Each plaque was enriched with 0.2ml of the bacterial host culture in 8ml of TSB at 37°C for 24h. After enrichment, the phage lysate was subjected to centrifugation at 8,000xg for 10min and then single plaque assay. Later, one plaque was selected for two more run of enrichment and plaque assay as describe above for phage purification. At the end, the phage lysates were filtered through 0.22μm syringe filter membrane and quantified by use of single layer plaque assay prior to further analysis.

### Transmission electron microscopy

Phage lysates were subjected to polyethylene glycol concentration using PEG kit (PEG, BioVision Inc., Milpitas, CA) according to manufacturer’s instruction, and morphology of the phage was examined by transmission electron microscopy (TEM). Briefly, an aliquot of 6μl of phage sample was applied on copper mesh PLECO grids (Ted Pella Inc., Redding, CA) and incubated for 1min at room temperature. The copper mesh grid containing phage sample was carefully blotted on Whatmann filter paper and subjected to negative staining by adding 8μl of 0.75% Uranyl acetate (Sigma-Aldrich, Darmstadt, Germany) for 30s incubation at room temperature. The specimen was then examined in a transmission electron microscope (FEI Tecnai G_2_).

### DNA extraction and PCR screening

After phage concentration by PEG, the phage lysates were subject to DNA extraction with commercial phage DNA extraction kit according to manufacturer’s instruction (Norgen Biotek Corp., Ontario, Canada). Detection of *stx* genes were investigated by use of PCR with 10μM each of *stx1* forward/ reverse primer pairs (5’-CATCGCGAGTTGCCAGAATG-3’/ 5’-AATTGCCCCCAGAGTGGATG-3’) and *stx2* forward/ reverse primer pairs (5’- GTATACGATGACGCCGGGAG-3’/ 5’- TTCTCCCCACTCTGACACCA-3’) [14]. PCR master mix (Promega Corp., Madison. WI) was added to the sample with a total reaction volume of 50μl, and thermal parameters for denaturing, annealing, and extension temperatures of *stx1* and *stx2* genes are 95°C, 56°C, 72°C and 95°C, 58.1°C, 72°C, respectively, for 28 cycles. The target amplicons were 119bp for *stx1* and 104bp for *stx2*. The PCR products were subjected to electrophoresis using 1.5% agarose gel with a 100bp DNA ladder at 100 voltages for approximately 2h and stained with 3x GelRed prior to visualization on the Alpha Imager UV gel box.

### Collection of environmental data

The information of rain precipitation and solar radiation were collected from the website of California Irrigation Management Information System (CIMIS). The rain precipitation data for each sample site were collected during 5 days ahead through the day of samples collection, and the solar radiation data was collected only on the day of sampling for statistical analysis. These environmental factors, rain precipitation and solar radiation, were included in this study to evaluate their effects on the prevalence of free STEC-specific phage because these were two of the major factors that attributed to the growth of produce.

### Data analysis

Statistical analysis was performed using JMP^®^ (Version 12.0.1, SAS Institute Inc., Cary, NC). One-way analysis of variance (ANOVA) tests were used to evaluate correlation between environmental factors (rain precipitation or solar radiation) and STEC-specific phage isolation. Student T test was used to evaluate different environmental impacts (human vs. agriculture) on the phage isolation.

## Results and discussion

This is the first study focusing on the prevalence of free STEC-specific bacteriophages at a pre-harvest produce area in Salinas, CA. In order to increase isolation of the free STEC-specific phages, both non-pathogenic and pathogenic STEC cocktails were used. Furthermore, the use of generic *E*. *coli* would also facilitate isolation of the phages harboring *stx* genes in this study. A previous study utilized non-pathogenic O157 strain to isolate Stx-phages from waste water and river water, and the findings showed that *stx2* was more prevalent than *stx1* in the phages isolated from the aquatic environment [15]. In addition, the authors revealed that the isolated Stx-phage were at low levels in their study. On the contrary, in current study, none of the STEC-specific bacteriophages were positive of either *stx1* or *stx2* gene. Therefore, there was less likely that the phages isolated in this study could serve as potential mobile genetic elements for transferring virulence genes in the environment [16]. The current results with no detection of *stx* genes in the phages could be due to low levels of fecal contamination around produce-growing environment in Salinas, which was correlated with the findings of previous research, in that the authors failed to track fecal contamination in Salinas area using male-specific coliphages as indicators due to scarcity of STEC strain isolation [12].

**Fig 2:**
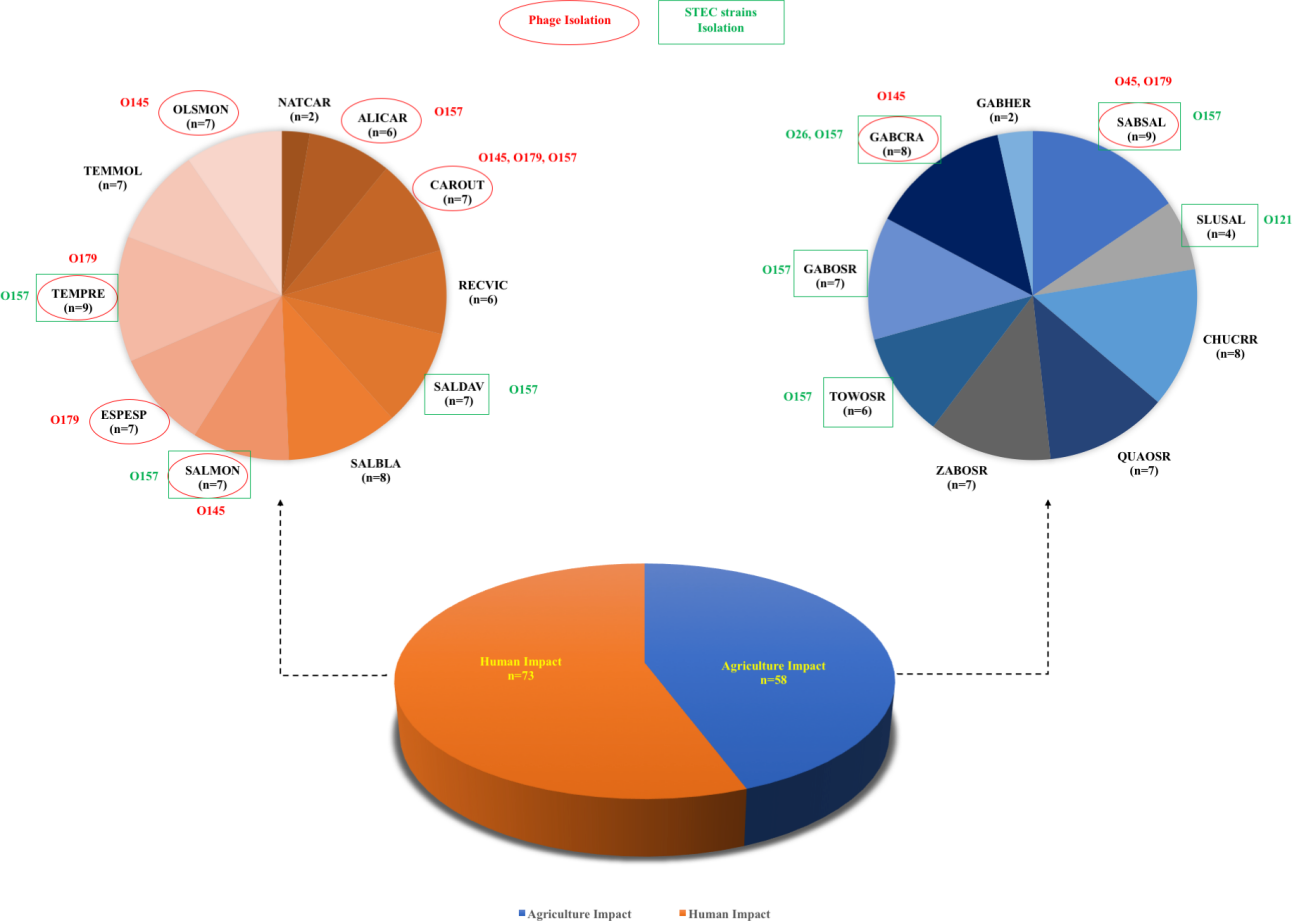
Summary of phage and STEC isolation data from water samples collected from Salinas, CA. The acronym is used for each sample site with the number of water samples collected. Red oval shape with red letter indicates isolation of STEC-specific phage, and green rectangular shape with green letters indicates STEC bacterial isolation.

In this study, a number of STEC-specific phages and STEC strains isolated from various sample sites in Salinas area are indicated in Fig 2 (131 samples from 20 watershed sites). A total of 9.9% (n = 13) of the water samples collected in this study were positive of free lytic bacteriophages against STEC strains, including serogroups of O45, O145, O157 and O179, and were distributed among 8 different watershed sites. The O145- and O179-specific phages were most frequently isolated (4 different sites) in this study, followed by O157-specific phages (2 sites), and O45-specific phages (1 site). Interestingly, both O145- and O179-specific phages were likely found in the area close to city where human impact was predominant; i.e., the site “CAROUT” was positive for three different STEC-specific phages (O145, O157 and O179). Although not statistically different, the results showed that the naturally occurring STEC-specific phages were more likely isolated in the human impact-associated area rather than in agricultural impact region (P>0.05). Additionally, the sample sites with isolation of O145-specific phages (GABCRA, CAROUT, SALMON, OLSMON) were either located in the same flow path (GABCRA & CAROUT) or affected by nearby merging river branch, indicating that the phages were likely to be disseminated through river flow. Similar phenomenon was also found on some of the free O179-specific phages positive sites (CAROUT, ESPESP, TEMPRE). On the other hand, though not subjected to analysis, more sample sites in the agriculture-impact area were positive of the STEC strains than those in human-impact area that could be due to cow calf operation or wild animal activities. Furthermore, O157 STEC strains were the most predominant bacterial isolates found in this study, both in areas with human impact and agriculture impact, and the river might also attribute to the spread of the pathogens. Although some sample sites were positive of both STEC strains and STEC-specific phages, it seemed that the sites with presence of STEC-specific bacteriophages did not have the STEC bacterial hosts isolated. The findings could be derived from the mitigating effects of these lytic bacteriophages against the STEC bacterial population because the spot test used at the early stage of the phage isolation showed that the phages were able to produce lysis zone against some of the top six non-O157 and O157 STEC strains selected in this study. Hallewell et al. studied the prevalence of *E*. *coli* O157:H7-infecting phages in the cattle that shed the O157 strains. The authors found the cattle that shed O157:H7 the most had lower prevalence of O157-specific phages, whereas the low O157:H7 shedder cattle had higher prevalence of the phages [17]. The antibacterial effect of the free STEC-specific phages isolated from either produce-growing environment or animal-associated habitats showed the potential of these free lytic phages as bio-control agents to prevent STEC contamination.

**Fig 3:**
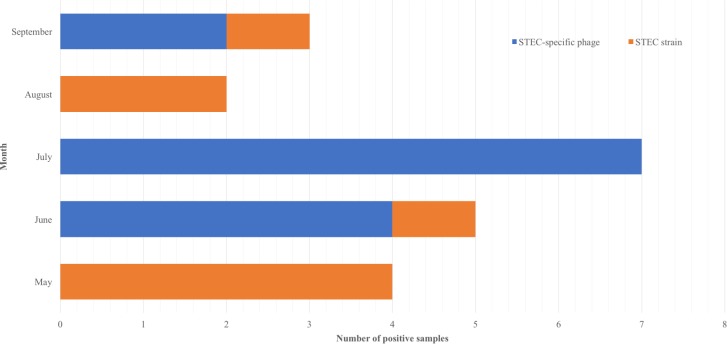
Samples with positive of STEC-specific phages and STEC strains throughout sampling period displayed by sample month.

In current study, the samples used for the isolation of free phages and STEC strains were collected from May to September 2016, in which covered late spring, summer and early fall seasons. The results showed that July had the highest overall prevalence of STEC-specific phages regardless of types of environment (human impact vs. agriculture impact) of the sample sites without any isolation of STEC bacteria, followed by June with the second highest prevalence of free phages (Fig 3). However, in May and August, STEC strains were frequently isolated from the sample sites, while no free phage was found. It was interesting to observe in this study that the more free STEC-specific phages were present, the less isolation of STEC strains was obtained during the same period of time. Ravva et al. evaluated the fecal contamination in Salinas area by use of male-specific coliphages, and their findings indicated that the DNA coliphages were predominantly isolated in summer time, which was consistent with our results regarding prevalence of STEC-specific phages [12]. In addition, a previous study evaluated the seasonal prevalence of O157 STEC and non-O157 STEC in Salinas area and found that overall prevalence of STEC strains was lower during summer and fall periods [11]. Their findings were correlated with our findings, in that higher prevalence of STEC-specific phage might account for the low prevalence of STEC bacterial isolates during summer time.

The results showed that, though not statically significant (P = 0.079), the sample sites with higher rain precipitation were likely to have higher prevalence of free phages (Table 2). This could be due to higher water levels by rain precipitation resulting in the spread of phages through affluent river flow all over the area, which was consent to the earlier observation that river flow may play an important part of facilitating phage dissemination. On the other hand, the current results also indicated that there was no significant difference (P = 0.717) between solar radiation and phage isolation, with the average radiation intensity of 476 Ly and 465 Ly at overall phage positive and negative sample sites, respectively (Table 2). Previous study indicated that the bacteriophage Lambda was resistant to UV radiation in a lab setting and it was able to remain viable under high-dose UV (300mJ/cm^2^) [18]. Although this study did not design to evaluate the environmental fitness to the bacteriophages, the current data likely suggest that the environmental factors, rain precipitation and solar radiation, had minimal effect on the prevalence of free STEC-specific phages.

**Table 2:** Effect of the environmental factors, rain precipitation and solar radiation, on the isolation of free STEC-specific phages from the overall sample sites.

Environmental factors^a^	Sample sites		P value
	Phage positive	Phage negative	
Rain precipitation (Inch±SD ^b^)	0.007±0.024^b^ A	0.003±0.008A	0.0791
Solar radiation (LY±SD)	476±341 B	465±111B	0.7169

^a^Least-squares means were calculated within each factor, and values within each factor with the same letter (A, B) are not significantly different (P>0.05).

^b^Data presented are mean ± SD (standard deviation).

**Fig 4:**
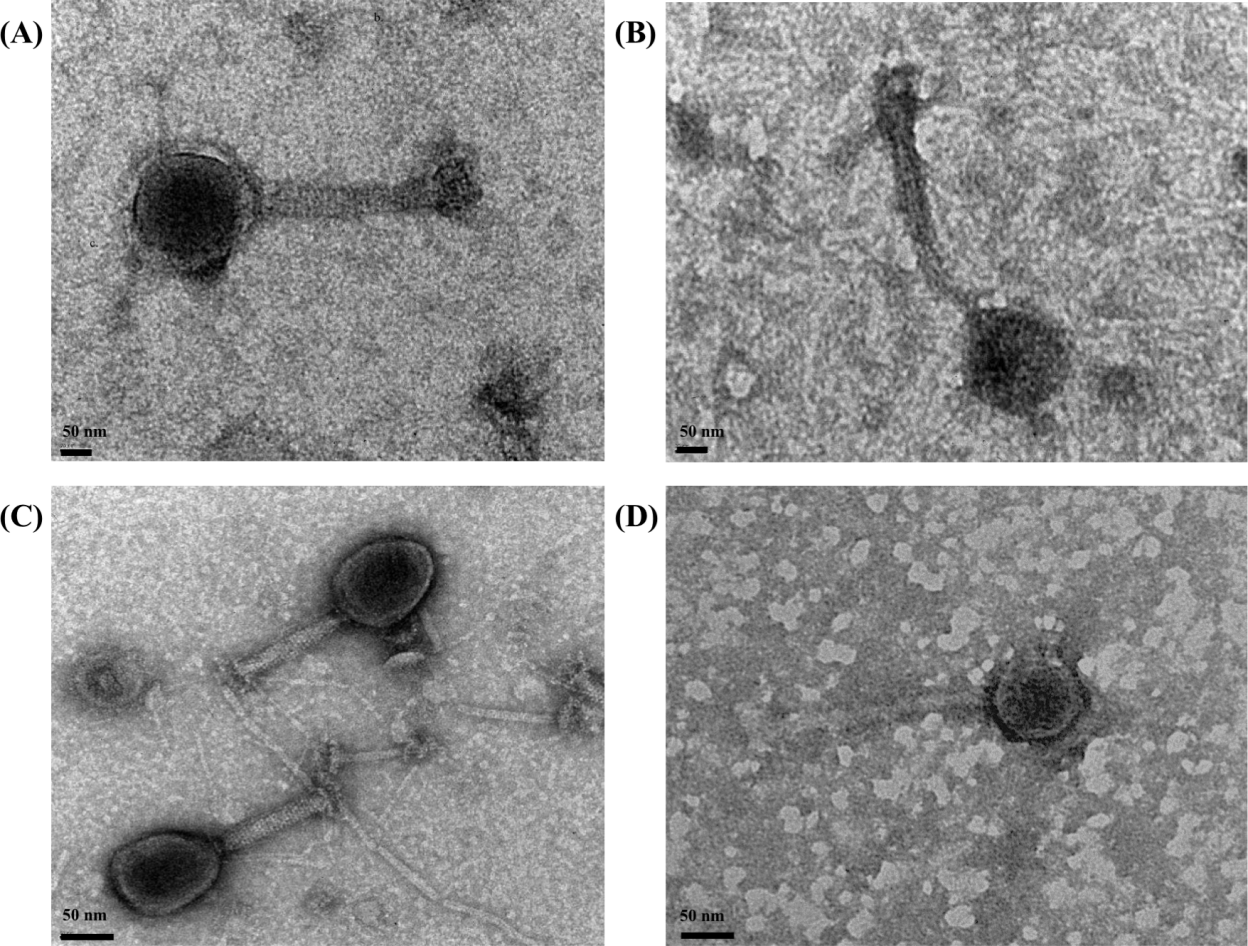
The morphology of the STEC-specific phages isolated from Salinas area. (A) O157-specific bacteriophage, (B) O145-specific bacteriophage, (C) O45-specific bacteriophage, (D) O179-specific bacteriophage.

Moreover, the electron microscopy showed that these STEC-specific phages had different morphologies belonging to *Siphoviridae* or *Myoviridae* family (Fig 4). Although phage morphology was not tightly correlated with specific serogroup of the host strains, the results revealed that both O45- and O157-specific phages that were isolated by use of generic *E*. *coli* cocktail were likely to be *Myoviridae* family, while O145- and O179-specific phages isolated by STEC cocktail were *Siphoviridae* family.

In conclusion, this is the first study to determine the prevalence of STEC-specific phages against serogroups of O26, O45, O103, O111, O121, O145, and O157 in pre-harvest produce environment in the US. The present study discovered STEC-specific bacteriophages, including serogroups of O45, O145, O157 and O179, which did not harbor *stx* genes. The findings suggest that the free STEC-specific phages were commonly isolated in the locations close to city or area with predominant human impact, and were likely disseminated from one place to another through river stream. The season, particular summer, contributed to higher prevalence of free STEC-specific phages in pre-harvest produce area in this study, even though rain precipitation and solar radiation did not have significant influence. Most interestingly, these results possibly suggest the mitigating effect of the free STEC-specific phages on its STEC bacterial hosts in the produce-growing area, i.e., the prevalence of lytic STEC-specific bacteriophages is negatively correlated with STEC strains in the pre-harvest produce environment. Further analysis is needed to evaluate effects of other environmental factors or the interaction of the environmental factors in long-term sampling plan.
